# Recent trends in non-invasive *in situ* techniques to monitor bacterial colonies in solid (model) food

**DOI:** 10.3389/fmicb.2015.00148

**Published:** 2015-03-06

**Authors:** María M. Lobete, Estefania Noriega Fernandez, Jan F. M. Van Impe

**Affiliations:** ^1^Flemish Cluster Predictive Microbiology in Foods, Leuven, Belgium; ^2^Chemical and Biochemical Process Technology and Control, Department of Chemical Engineering, Katholieke Universiteit Leuven, Leuven, Belgium

**Keywords:** bacterial colonies, growth dynamics, solid food (model) system, image analysis, food structure, optical density, microcalorimetry

## Abstract

Planktonic cells typically found in liquid systems, are routinely used for building predictive models or assessing the efficacy of food preserving technologies. However, freely suspended cells often show different susceptibility to environmental hurdles than colony cells in solid matrices. Limited oxygen, water and nutrient availability, metabolite accumulation and physical constraints due to cell immobilization in the matrix, are main factors affecting cell growth. Moreover, intra- and inter-colony interactions, as a consequence of the initial microbial load in solid systems, may affect microbial physiology. Predictive food microbiology approaches are moving toward a more realistic resemblance to food products, performing studies in structured solid systems instead of liquids. Since structured systems promote microbial cells to become immobilized and grow as colonies, it is essential to study the colony behavior, not only for food safety assurance systems, but also for understanding cell physiology and optimizing food production processes in solid matrices. Traditionally, microbial dynamics in solid systems have been assessed with a macroscopic approach by applying invasive analytical techniques; for instance, viable plate counting, which yield information about overall population. In the last years, this approach is being substituted by more mechanistically inspired ones at mesoscopic (colony) and microscopic (cell) levels. Therefore, non-invasive and *in situ* monitoring is mandatory for a deeper insight into bacterial colony dynamics. Several methodologies that enable high-throughput data collection have been developed, such as microscopy-based techniques coupled with image analysis and OD-based measurements in microplate readers. This research paper provides an overview of non-invasive *in situ* techniques to monitor bacterial colonies in solid (model) food and emphasizes their advantages and inconveniences in terms of accuracy, performance and output information.

## EFFECT OF SOLID STRUCTURES ON THE GROWTH OF BACTERIAL POPULATIONS

Microbial behavior is determined by structure as much as by environmental conditions and the chemical composition of the media (Robins and Wilson, 1994; Wilson et al., 2002; Antwi et al., 2006, 2007; Theys et al., 2008). In solid structures, substrates diffuse into the colonies and metabolic products outside, triggering concentration gradients in and around the colony, that concern cells availability to oxygen and nutrients (Wimpenny and Coombs, 1983; Wimpenny et al., 1995; Walker et al., 1997; Malakar et al., 2000, 2003; Kabanova et al., 2013). The outwards diffusion of metabolic acids in bacterial colonies gives rise to pH gradients, causing a potential degree of biochemical heterogeneity within and around the colony cells (Walker et al., 1997; Malakar et al., 2000). Studies focused on oxygen penetration’s depth and its diffusion through the matrix have revealed to play also a key role on bacterial colonies growth, regarding their surface or submerged character (Wimpenny and Coombs, 1983; Peters et al., 1987; Robinson et al., 1991). Solid structures has also been reported to affect certain bacterial characteristics, e.g., cell development, morphology, membrane permeability, surface tension, osmotic pressure and metabolism, as well as cell viability and physiological state (Dervakos and Webb, 1991; Wilson et al., 2002; Meldrum et al., 2003). Some authors stated that cell immobilization in a solid environment can also generate changes in cell susceptibility to antimicrobial compounds (Walker et al., 1997; Malakar et al., 2000; Skandamis et al., 2000; Dens and Van Impe, 2001) as well as acid tolerance responses (Malakar et al., 2000; Antwi et al., 2007).

Therefore, it seems of outmost importance to study colony behavior in solid structures, since most of the studies in literature have focused on planktonic growth in liquid systems. In fact, trends in predictive microbiology are moving toward a more realistic approach of microbial behavior in structured food products, including the study of surface and submerged colonies. This work gives an overview of the different available techniques to characterize colony growth in solid model food systems.

## STUDYING BACTERIAL COLONIES IN SOLID MODEL FOOD SYSTEMS

### INVASIVE TECHNIQUES

To date, several approaches have been developed for a better understanding of bacterial colony behavior in solid systems. Most traditional measuring techniques provide information of overall microbial populations through invasive procedures, like viable plate count and microelectrodes. Traditional techniques enable growth dynamics estimation, the measurement of environmental conditions, etc. Hereunder are compiled some representative achievements in this field.

To study colony behavior at laboratory scale, bacterial colonies are often obtained from the inoculation of different solid culture media prepared by adding different gelling agents. Agar and gelatine are widely used in food microbiology, although their relevance is restricted to products with gelled microstructure like frankfurters (Mertens et al., 2009). In order to cover a wider range of model food systems, some authors assayed with different hardening agents such as xanthan gum, Carbopol, κ-carrageenan (Mertens et al., 2009, 2011; Noriega et al., 2010; Boons et al., 2013a) and Maxiren 180 (DSM Food Specialities, France), a coagulant agent used by Jeanson et al. (2011) for a model cheese. Studies on real solid food have also been performed, e.g., Katsaras and Leistner (1988) described for the first time the growth of submerged bacteria in fermented sausages; Noriega et al. (2010) described the role of the food structure in bacterial colonization of poultry products. The study of bacterial colonies also requires an adequate experimental setup to hold the solid structure in which colonies will be cultured, e.g., plastic or glass petri dishes and conical tubes. Special attention draws the gel-cassette system designed by Brocklehurst et al. (1995) at the Institute of Food Research (IFR, Norwich, UK). This system consists of a frame sealed with gas-permeable plastic film that holds the inoculated gelled media between two PVC transparent windows. The gelled media needs a separate preparation in order to be inoculated and introduced in the system before solidifying. Once the cassette is filled, it is sealed with the plastic films, being then ready for incubation. Sampling for viable count experiments from the gel-cassette requires blending the media to take an aliquot; while for colony observation it can be directly couple to a microscopy device. The gel-cassette is widely used to develop either submerged or surface colonies and has helped assessing overall population dynamics of several pathogenic bacteria, e.g., *Salmonella typhimurium* (Brocklehurst et al., 1995), *Listeria monocytogenes* (Meldrum et al., 2003) and mixed bacterial co-cultures (Tsigarida et al., 2003).

Among the traditional techniques to determine bacterial growth in solid structures, viable plate count remains as the most commonly used procedure. This technique generally entails the sampling of an aliquot from the inoculated system under study, its homogenization and dilution. Finally, the sample is spread on the corresponding agar plates for the following viable counting. This technique has been used to determine the overall growth dynamics of bacterial populations and thus, to elucidate the potential effect of different environmental factors. Brocklehurst et al. (1995) assessed the effect of transient temperatures on the growth dynamics of *S. Typhimurium*. Noriega et al. (2013) used global viable counts to determine the effect of medium structure on the sublethal injure of *S*. *Typhimurium*, *L. monocytogenes* and *Escherichia coli* colonies. In Skandamis et al. (2007) viable plate count was compared with a non-invasive technique to study the effect of temperature and pH on the growth of *E. coli* colonies cultured in the gel-cassette. Although this methodology is widely applied in the study of bacterial colonies, viable plate count gives information of overall population dynamics, but not from the individual colonies; moreover, it requires a tedious, time consuming and costly work (Guillier et al., 2006; Jeanson et al., 2011; Mertens et al., 2012; Koutsoumanis and Lianou, 2013). Additionally, the heterogeneous behavior among individual cells is driving researches to study cell colonies individually, instead of as a whole population.

As previously described, colony growth in solid systems is affected by several diffusion limitations responsible for gradients of pH, oxygen, metabolites, etc. For a deeper insight in these localized gradients, an invasive technique based on direct measurements with microelectrodes has been developed. Wimpenny and Coombs (1983) published the first work in which microelectrodes were applied to measure oxygen penetration. Colonies of *Bacillus cereus* were cultured on the surface of tryptone soya broth agar (TSBA) contained in petri dishes and oxygen measurements were performed with an oxygen-sensitive microelectrode mounted on a prior micromanipulator. On the other hand, it has been reported the potential inaccuracy in the use of microelectrodes, due to oxygen leakage around the electrode and poisoning tip (Tammam et al., 2001). Microelectrodes have also been applied for measuring pH gradients, e.g., Walker et al. (1997) described the pH gradients within and around surface colonies of *S. Typhimurium* grown in an adapted gelatin cassette system. This technique is usually combined with viable plate count, so that, information about colony growth assist in interpreting the obtained measurements. Nowadays, few tools are available to monitor and identify the metabolic release patterns of growing microbial colonies. Among them, matrix-assisted laser desorption/ionization-time of light (MALDI-TOF) imaging mass spectrometry (IMS) has recently been proposed as a valuable source to detect and to (2D) and (3D) visualize the distribution of metabolites produced by microbial colonies (Gonzalez et al., 2012; Fang and Dorrestein, 2014). Through IMS, the whole bacterial colony is examined including the surrounding agar medium, defining a raster composed of greater that 1000 laser points of data collection, increasing thus the likelihood of detecting unique, discrete ion distribution patterns and hidden molecular phenotypes (Gonzalez et al., 2012). In addition, its combination with MALDI-TOF imaging unravels to visualize the spatial distribution of the detected compounds.

The study of microbial colonies and their peculiarities has also been extended to microbial identification and characterization. It is worth to mention certain techniques that although invasive, approach microbial colonies from an individual point of view. For instance, Fourier transform infra-red (FT-IR) spectrometry provides fingerprint-like patterns of microorganisms that enable to identify different species and strains due to their specific spectra. The efficiency of this method has been demonstrated for several bacterial species such as *Staphylococcus*, *Streptococcus*, *Clostridium* and *Legionella* (Helm et al., 1991), among others. Thi et al. (2003) assessed an improvement of this technique by coupling the FT-IR spectrometer with a light microscope and obtain microscopic spectra that provide information on the number, size and shape of the micro-colony cells. Furthermore, this technique was extended in Thi and Naumann (2007) to study the spatial heterogeneity of cell growth within macro-colonies. Cells were grown in agar under optimal conditions in order to get macro-colonies with a diameter of about 3–5 mm. These macro-colonies were excised from the agar, frozen at –70^°^C and cryosectioned in slices of 20 μm for further FT-IR mapping measurements. Obtained spectra revealed a substantial spatial heterogeneity of cell composition among the microorganisms studied (*Legionella bozemanii*, *Bacillus megateriuma* and *Candida albicans*).

Invasive techniques have clearly contributed to increase the knowledge about bacterial colonies behavior; however, their invasive character, the subsequent sample discard and the lack of individual information gathered at the colony level are disadvantages to be overcome. In order to collect more accurate information and fundamental knowledge, non-invasive analytical techniques focused on mesoscopic and microscopic assessments of colony growth, are replacing the traditional ones (Mertens et al., 2012).

### NON-INVASIVE AND *IN SITU* TECHNIQUES

As previously described, most studies involving bacterial colonies are based on colony estimation of overall cell population dynamics in solid systems; however, an individual approach is required for a deeper insight in single colony behavior. Latest trends are moving toward the development of non-invasive techniques that enable to monitor bacterial colonies without altering their environment or subsequent sample discard. These techniques offer the advantage of obtaining quantitative data, faster and more accurately. Hereunder some of these novel techniques are grouped by the applied principle, which to date are mainly imaging techniques, optical density (OD) and microcalorimetric techniques. Nevertheless, the transition from traditional to novel techniques is gradually occurring since the latter also present some limitations. Under this situation, it can be observed that in many of the works here reviewed, novel techniques are either combined or compared with traditional ones, such as viable plate count.

#### Imaging techniques

The use of microscopy-based methods enables to monitor growth of bacterial colonies in real time and to estimate different parameters related to colony evolution. This can be obtained through a further analysis of the obtained images. These methods are gaining interest in the field of food microbiology, since image analysis programs and automation software tools make data processing more feasible (Skandamis et al., 2007). Hereunder are some of the most applied techniques in this field detailed.

***Light microscopy and digital camera coupling.*** Direct colony observation through a light microscope provides a useful tool to monitor certain colony parameters such as the colony radius and their area evolution on the surface. The combination of a light microscope with a digital camera offers the possibility to immortalize the visualized image for further process and study. Several studies based on these techniques have been conducted to estimate colony dynamics, i.e., growth rate and lag phase, which are generally the most representative parameters to be identified (Guillier et al., 2006; Rasch et al., 2007; Skandamis et al., 2007; Koutsoumanis and Lianou, 2013). With this aim, Guillier et al. (2006) introduced a new method to estimate and compare individual lag times of either stressed or exponentially growing colonies of *L. monocytogenes*. Obtained results were afterwards compared with the individual lag time distributions observed in broth. In this work, the setup for surface colony development was agar plates, on which the colonies were monitored with a digital camera PixelFly (Photon Lines) and an automatic image processing system. Initially, suspensions of *L. monocytogenes* cells were disposed with a spiral plater on plates of tryptone soya agar enriched with yeast extract (TSAYE). During incubation, images were taken at constant time intervals of 10 min. The imaging set-up for this study consisted of a circular motorized platform OptoSigma (Photon Lines, Marly-le-Roy, France), that was coupled with the digital camera and enabled to study four plates simultaneously. While the colonies were growing, the intensity of the pixels in the obtained images was also increasing and this increment was then related to the lag phase. In this context, the time required for a colony to reach a surface threshold is considered proportional to the lag time of the cell initiating the colony, as also assumed for turbidity-based methods. This work presents a valuable method for studying colony growth dynamics since results showed means and standard deviation values similar to those obtained in broth, as well as no clear difference between liquid and solid conditions.

One of the main systems used for obtaining and visualizing submerged and surface colonies, is the gel-cassette. The gel cassette set-up was already widely used to estimate population growth dynamics in solid systems and it was at the beginning of the 21st century when its specific configuration with two transparent windows, inspired the coupling with microscopy techniques, enabling thus *in vivo* colony visualization. Skandamis et al. (2007) applied this combination of devices, a light microscope coupled with a digital camera and the gel-cassette to determine the effect of temperature and pH on the growth rate of *E. coli* O157:H7 colonies. The gel-cassette system was aseptically filled in with brain heart infusion agar (BHIA) adjusted at different pH values and then inoculated. Afterwards, it was coupled to a microscope with a high-sensitivity SSC-DC50AP digital camera (Sony Corp, Tokyo, Japan) that allowed to capture images of the growing colonies. The evolution of the colony surface was monitored with 40 × objective lens from a light microscope and expressed in log_10_ transformed pixels. Surface area data were used to estimate maximum growth rates (*μ*_max_)_A_ by applying the model of Baranyi and Roberts (1994). The observed (*μ*_max_)_A_ values were fitted to a quadratic secondary model to express the natural logarithm of the (*μ*_max_)_A_ as a function of temperature and pH. Based on a correlation between the observed (*μ*_max_)_A_ and the observed viable count-based *μ*_max_ (*μ*_max_)_VC_, the predicted (*μ*_max_)_A_ values were transformed into predicted (*μ*_max_)_VC_. This model was validated by graphical comparison of the predicted (*μ*_max_)_VC_ with published (*μ*_max_)_VC_ values from *E. coli* O157:H7 in broth and different foods, as well as by mathematical means, i.e., the bias and accuracy performance indices (Ross, 1996). These data were gathered from *ComBase* database. Results showed that the model performed well with published responses of *E. coli* O157:H7 in broth, although a poorer agreement of the model was obtained from the correspondent responses of *E. coli* O157:H7 in food. It can be concluded that the use of (*μ*_max_)_A_ is an adequate alternative in the development of predictive models to describe the (*μ*_max_)_VC_ and a potential alternative to traditional viable plate count.

A further step in surface colony image monitoring with a light microscope was presented by Koutsoumanis and Lianou (2013). This research evaluated image series obtained at short time intervals, sequentially compiled and transformed into a video. The aim of this study was to assess the heterogeneity in the growth dynamics of micro-colonies emerging from single cells of *S. Typhimurium* investigation. Bacterial colonies from individual cells were grown on TSA disposed on a glass slide, which was covered with a coverslip. This set-up allowed to directly observe under the z-motorized microscope (Olympus BX61; Olympus, Tokyo, Japan), equipped with a high resolution device camera. Images were obtained with phase-contrast time-lapse microscopy and compiled to create the video. This video was a useful tool to characterize the cells in terms of division times, cell length and growth kinetics. Results showed a high heterogeneity in the growth behavior if individual cells are compared. This heterogeneity became higher for those cells that gave rise to small colonies. Generally, the experimental error from the application of monitoring methods, remains as the main constrain at describing colonial behavior. Time-lapsed microscopy method described in this paper, eliminated this limitation since it allowed to count each cell with time in each micro-colony.

***Fluorescence and confocal laser scanning microscopy.*** Fluorescence microscopy technique is based on detecting the emitted fluorescence by fluorescent molecules. The excitation of these molecules is followed by an emission of a band of light with a specific wavelength, being the excitation-emission spectra specific for each molecule. The use of fluorophores allows to stain target components such as, proteins, oil droplets, carbohydrates, etc., and thus, to visualize them. Several works have been presented to study bacterial colonies by applying this principle.

Malakar et al. (2000) presented one of the first works involving gel-cassette with a fluorescence microscope to monitor bacterial colonies and experimentally confirming the presence of pH microgradients within and around them. In this study, colonies of *Lactobacillus curvatus* were grown in De Man, Rogosa, Sharpe (MRS) broth with bovine gelatine as gelling agent and supplemented with Oregon Green^™^, a fluorescent dye which intensity was linearly related to a pH range; once prepared the mixture, it was aseptically introduced in the gel-cassette system for further analysis. Subsequently, a fluorescence microscope (Nikon, Surrey, UK) with the following filters, excitation (440 ± 10 nm, 510 ± 11.5 nm), emission (535 ± 11.5 nm) and a dichroic mirror (540 nm, long pass) was set up with the gel-cassette system and serial measurements of fluorescence intensity in the inoculated media were taken. These images were obtained after focusing on a defined plane between the bottom and top of each cassette-window and exciting this area with defined light and wavelengths. Furthermore, light transmission images were taken in order to determine the colony radius, once they were visible and put into focus. By representing the measured radius and pH values, 2D pH maps were elaborated. Finally, the superimposition of the pH maps with the transmission images, helped to reveal the pH profiles in and around the colony. As the authors stated, this technique was the first approach that allowed to monitor colony growth non-invasively, as well as to reinforce the concept of pH gradients in solid systems, being more representative of what happens in a colony in real time.

CLSM works on the same principle as fluorescence microscopy although it presents certain advantages, e.g., better resolution images due to the pinhole that eliminates out-of-focus light and the possibility to observe thicker samples, making optical sections at different depths that enables to reconstruct a 3D-image. This device, coupled with the gel-cassette was applied by Jeanson et al. (2011) to study colony size and distribution as a function of the inoculum level in a model cheese. In literature, has often been object of study the effect of the initial microbial load on colony size and distribution. High initial inoculum levels promote small colonies closely located; in contrast, low initial cell densities lead to large colonies separated by long distances (Malakar et al., 2002, 2003; Theys et al., 2009). As a consequence, inter- and intra-colony interactions appear, affecting bacterial behavior. Inter-colony interactions are typically found between small colonies separated by short distances in which the fast diffusive transport has been described as comparable phenomena to convection in broth (Malakar et al., 2002, 2003). In contrast, intra-colony interactions are dominant in large colonies formed from a low inoculation levels. In this scenario, diffusion becomes a constraint since it is not able to cope with the demands of the cells situated in the center of the colony; as a consequence, this delayed molecular transport within the colony triggers the interaction between the inner cells (Malakar et al., 2002, 2003). In this context, the work of Jeanson et al. (2011) aimed at describing this phenomenon in a cheese matrix with immobilized bacteria whose metabolism is the main responsible for its ripening. The model cheese matrix was developed at laboratory level with the retentate from milk ultrafiltration (UF) and coagulant agent Maxiren 180 (DSM Food Specialities, France). This model system was afterwards introduced in a gel cassette and inoculated with different levels of *Lactococcus lactis,* in order to observe the bacterial distribution under the CLSM. The selected strain produces a green fluorescent protein (GFP) that can be linked to the metabolically active state of the cells and therefore, observed under the microscope. Several CLSM photographs of the fluorescent colonies were taken aiming at characterizing their spatial distribution in the model cheese. This distribution resulted to be random in the whole structure, independently of the inoculum level. Nevertheless, the initial density was found to determine the spatial separation between the colonies, as previously reported by Malakar et al. (2003). Traditional viable plate counting was also carried out, confirming that independently of the initial inoculation level, the final stationary population remains equal. Besides colony size and distribution, Floury et al. (2013) used CLSM coupled with the gel-cassette system to study the potential porosity of the *L. lactis* colonies to certain compounds in a cheese model system. As above described, cheese ripening depends on the metabolism of immobilized bacteria. Thus, cheese ripening can be affected by diffusional limitations of nutrients and metabolites within and around the colonies. For this case study, colony porosity to different sizes of dextran molecules (from 4.4 to 155 kDa) was assessed in the model cheese system and in M17-agar. Both media were aseptically prepared, inoculated and disposed in mini gel-cassette systems for directly observation under CLSM. For the porosity observation, the chosen dextrans were fluorescently labeled with Rhodamine B isothiocyanate (Sigma Aldrich, Saint-Quentin Falavier, France) and viable and total bacterial counts were performed with the LIVE/DEAD^®^ Bacterial Viability Kit (BacLigh^™^, Molecular Probes, Invitrogen). Obtained images were also used to study the different morphologies of the colonies depending on the media; while in the model cheese they were spherical, colonies grown in M17-agar showed under the same growing conditions, a lenticular shape.

Besides the gel-cassette, other setups like chambered cover glass are applied for CLSM studies. Jeanson et al. (2013) used CLMS with CoverWell imaging chambers (Sigma-Aldrich, Quentin Fallavier, France) to assess the pH around colonies of *L. lactis* inoculated in the cheese model system with different inoculum levels. Bacterial colonies were visualized with the fluorescent dye SYTO-9 (Molecular Probes, Invitrogen, Villebon-sur-Yvette, France), which was excited at 488 nm and detected at 515 ± 15 nm. This visualization confirmed that the low inoculum levels gave rise to bigger colonies. A pH sensitive probe C-SNARF-4F (Molecular Probes, Invitrogen, Villebon-sur-Yvette, France) was used to monitor micro-pH around the colonies. This C-SNARF-4F was excited at 543 nm and detected at 590 ± 15 nm and over 650 nm, enabling afterwards to calculate the ratio (*R*) from these two peaks. Measurements were taken along 100 μm from the edge of the colony at 10 μm steps from opposite sides of the colony. In order to correlate *R* values with pH, macro-pH measurements were performed with a pH meter (Inlab pH level 1; WTW, Germany) and pH electrode (Sebtix 41; WTW, Germany) in flasks containing the inoculated model cheese and incubated under the same conditions as the imaging chambers. Results showed that lower inoculum levels yielded to lower acidification kinetics; however, from the micro-pH measurements around the colonies, no microgradients were identified independently of the size of the studied colonies. The absence of these microgradients reveals, for the first time, that the pH perceived by the cells in a model cheese while acidification, is actually the macro-pH. Boons et al. (2013b) also used CLSM with imaging well chambers (Nunc Lab-Tek (USA) chambered borosilicate coverglass system) to determine the effect of the media composition and microstructure. This paper describes for the first time the behavior of *E. coli* in a heterogeneous protein–polysaccharide solid media with CLSM. The main novelty in this work was to use a gelatine–dextran mixture at different concentration ratios, which led to different microstructures characterized by a phase-separation, affecting thus bacterial behavior. This phenomenon was observed in the system thanks to the Rhodamine B, which enabled gelatine phase visualization. Initially, increasing gelatine concentrations promoted the transition from a homogeneous to a heterogeneous structure in which dextran spheres appeared in the gelatine matrix. However, if the gelatine phase concentration was also increased, a phase inversion occurred and gelatine spheres appeared in the dextran matrix. This phase inversion induced *E. coli* colonies to grow as diffuse strings instead as defined spheres. This morphological characterization was observed thanks to in the Venus fluorescent protein included in the *E. coli* strain, which expression enabled to visualize the bacterial cells with CLMS. In spite of these morphological changes, colony development occurred exclusively in the dextran phase. The same imaging technique was applied in Boons et al. (2014), although viable plate counting was also performed to estimate the microbial dynamics of *E. coli* in the heterogeneous microstructure, in order to decipher relation between micro- and macro-scale results.

***Imaging techniques for identification.*** Besides monitoring microbial growth, imaging techniques are also a useful tool to study specific microbial colony characteristic, such as strain identification. In this context, label-free bacterial colony phenotyping technology, also called bacterial rapid detection using optical scattering technology (BARDOT), has shown promising results in bacterial identification and classification. Kim et al. (2014) presented a theoretical model in order to explain the underlying mechanism of speckle formation by the colonies from *Bacillus*. Laser-based optical sensor interrogated the entire volume of the colonies in order to gather information that could be encoded in the far-field scatter patterns. This technique was also assessed by Bae et al. (2010) on bacterial colonies of *S. Montevideo*, *L. monocytogenes* and *E*. *coli*, obtaining results that correlate colony morphology and growing characteristics with characteristic diffraction patterns.

It can be concluded that a wide range of applications are available for studying bacterial colonies with non-invasive imaging methods, although many authors still include traditional invasive techniques in their studies, like viable plate count, to enumerate total cell population. Experimental setups based on microscopy devices coupled with high resolution cameras and, e.g., the gel-cassette system, provide a valuable and promising tool to monitor individual cell colonies. However, their routine application in food microbiology remains a challenge since they also have some limitations.

For instance, the use of the gel-cassette limits the number of experimental conditions that can be assessed simultaneously (Mertens et al., 2012), as well as the tedious required work for the data analysis. These limitations point out the need for further studies to optimize available non-invasive methods to monitor colony behavior.

#### Alternative methods

Optical density measurements have been traditionally applied as an indirect method to monitor individual cell growth, which is related to the media absorbance evolution. Based on Beer’s law, OD is proportional to microbial concentration, as described by Koch (1981). In diluted suspensions, most bacteria have nearly the same absorbance per unit of dry weight concentration, independently of cell size. This method appeared as an alternative to more laborious and time consuming techniques such as viable plate count. OD-based methods represent a useful tool for growth parameter estimation due to its non-destructive character and its ability to fast provide high-throughput data; furthermore, it is not expensive and relatively easy to automate in comparison to other classical methods (Mertens et al., 2012). Several authors have applied this method to address microbial growth of freely suspended cells (McClure et al., 1993; Dalgaard et al., 1994; Nerbrink et al., 1999; Dalgaard and Koutsoumanis, 2000; Métris et al., 2006). Traditionally, measurements were taken one by one in spectrophotometers, requiring hard work and limited number of samples; however, this technique has evolved and more advanced devices, such as the Bioscreen C^®^, Versamax^®^, etc., have appeared in the market. These latest set-ups are provided with reading chambers for microtiter plates that contain numerous wells; moreover, temperature can be programmed as well as the number of measurements and the interval time. These characteristics enable to simultaneously study different environmental conditions due to the multiple and independent wells per plate and a high-throughput non-invasively data collection. For a direct data processing, several software packages have been developed that can be coupled with the mentioned experimental devices.

OD-based techniques have been recently applied for colony growth characterization. Mertens et al. (2011) used the SpectraMax M2^e^ microplate reader to assess the effect of a solid environment on the growth/no growth (G/NG) boundaries of *Zygosaccharomyces bailii,* in comparison to liquid media. This work supposed the first approach of an OD method to study microbial colonies. In this work, the structured model system was based on Carbopol and xanthan gum as gelling agents, which were independently tested and, in the case of Carbopol, prepared at different concentration ratios. Rheological measurements were applied to characterize the different media based on the Carbopol concentration. From this measurements it was shown that higher concentration levels gave rise to highly structured systems and those with intermediate levels of Carbopol induced low structured systems. After preparation, the different media were inoculated and distributed in the 48-well microtiter plates. Prior to inoculation, a preculture of *Z. bailii* was obtained under optimal growing conditions and serially diluted to reach an inoculation level of 5 × 10^4^ CFU/mL. Due to the planktonic character of the cells in the preculture, the last dilution before inoculation was carried out in the same structured model system used afterwards. This specific model system, developed by Mertens et al. (2009), aims at obtaining similar conditions to those found in acidic sauces like ketchup, which is commonly spoiled by *Z. bailii.* Including these compounds as gelling agents supposed a novelty in the area, as well as the use of *Z. bailii* in solids, since the majority of studies to date have focused on agar or gelatine and on pathogenic bacteria, respectively. Once the microtiter plates were filled in with the inoculated media and the equipment conditions settled, measurements were programmed to be taken at regular time intervals. For the experiments with planktonic cells, OD was determined at one single point of each well in the microtiter plate; however, in the case of the solid structure, the scan method was performed by measuring every well at nine different positions. Afterwards, obtained values were used to plot OD curves. Results showed that yeast colonies development depended on the structured degree of the media. In one hand, intermediately structured Carbopol media led to numerous and homogeneously distributed colonies, easily identified with a shift in the OD-based growth curves. On the other hand, highly structured Carbopol media limited colony formation to certain environmental conditions. For environmental conditions close to the G/NG boundaries, between none and small few colonies appeared and remained mainly undetectable. This supposes a limitation of the method since just one single cell can yield a colony, risking food safety and quality. Authors concluded that it would be more realistic to independently consider the nine measurements taken at different positions of each well, instead of as mean values, for more accurate identification and definition of colonial behavior. This method was improved in Mertens et al. (2012) to study the colony growth of *E. coli* at optimal temperature (30^°^C). As in the previous study, the area scan method was applied by measuring OD at nine different points of each well; however, a much higher resolution was applied this time in order to characterize colony dynamics and measuring points were individually considered. This study was performed on submerged and surface individual colonies after inoculating the wells with an initial level of 1–2 cells, which was achieved after performing the adequate decimal dilutions from the preculture. To obtain surface colonies, the spot inoculation method was carried out by pipetting a single drop from the preculture on the agar surface. OD measurements were performed with a FilterMax^™^ F5 microplate reader with Multi-Mode Analysis software at a wave length of 590 nm. Results provided the necessary information to calculate the colony area by establishing 100 pixels as the minimum number to consider an object a colony. Authors reported a correspondence of this value with approximately 6 log (CFU/colony). Through the scan method the evolution of the area of colonies was followed, which was linearly expanding with time and showed a similar trend between the independent colonies at early stages. At an individual level, an OD increment at the center of the colonies showed an increase in their height. The length of the experiments lasted for 23 h, although this time is not the absolute limit of growth for a colony, the limited dimensions of the individual wells determined the experiments duration. The authors concluded that besides the limitations of the method, OD is an easy-to-use and flexible technique for monitoring bacterial colonies.

OD-based techniques provide many advantages for studying bacterial colonies, but there are still some limitations that it is necessary to overcome such us, the extended time required for the adequate data analysis, the uncertainty of the measured cell state (live, dead, sublethally injured, etc.), as well as some discrepancies between OD and viable count methods.

Another novel non-invasive technique to monitor bacterial colonies is based on microcalorimetric measurements. As defined by Braissant et al. (2010), isothermal calorimetry measures the heat flow of biological processes, which is proportional to the rate at which a chemical or physical process takes place. Microcalorimeters are the specific devices used in this field that measure heat flow of less than a micro-watt. The high sensitivity of the microcalorimeters favors their application in microbiology, since they can determine the heat released by bacterial cells while growing. This heat is represented as a function of time and the obtained curves are called calorimetric thermograms or power–time curves. Information gathered from this curves has been quantitatively correlated with biomass generation, changes in the number of cells, uptake oxygen, or substrates, etc., (Birou et al., 1987). This method presents several advantages for microbial studies, like the little preparation of the samples, non-invasive character (Braissant et al., 2010) and a continuous signal in real time. As a consequence, the applications of this method have been widely spread in different fields, more specifically for clinical (Trampuz et al., 2007), environmental and food microbiology (Gram and Sögaard, 1985; Alklint et al., 2005).

In the last years, microcalorimetric methods are drawing attention for studying bacterial colonies. The application of this technique to monitor and describe the growth of bacterial colonies in solid model systems, was presented for the first time by Kabanova et al. (2012). The main objective of this work was to assess the growth of individual colonies of *L. lactis* of different sizes. To obtain colonies of different sizes, serial inoculation levels were prepared from 10^0^ to 10^6^ CFU/mL by serially diluting the corresponding preculture. Colonies were grown in carbohydrate restricted medium (CMR) agar, which was initially inoculated and afterwards aseptically introduced in the specific ampoules for the microcalorimetric analysis. This analysis was carried out in a multi-channel thermal activity monitor TAM III, in which the heat is measured with a heat conduction calorimeter. From the resulting power–time curves different growth phases were identified, i.e., lag, exponential and deceleration or stationary phase (Kabanova et al., 2012, 2013). Maximum growth rate showed a dependency on the inoculum level, i.e., low inoculum levels led to higher values than those obtained from high inoculum levels. Although methods based on calorimetry allowed to study bacterial colonies non-invasively, the estimated growth dynamics gave information of the overall population. Thus, in Kabanova et al. (2012) the dimensions of the colonies were measured with a microscope Zeiss Axiovert 200 M coupled with AxioCam MRc5 camera and Zeiss AxioVs40 V45.0.0 software. Colonies up to 10^4^ CFU/mL showed a lenticular shape. This morphology was related to the agar gel structure and the size of the pores in which colonies grow. Once the colony grew over the pore size, the bonds between chains of the gel agar were broken, triggering a lenticular colony formation. Furthermore, this microscopically analysis revealed a homogeneous distribution of bacterial colonies in the media with the same characteristics as reported by Jeanson et al. (2011).

It can be concluded that microcalorimetry is a powerful technique to collect information of cell growth in solid (food) systems. Moreover, Kabanova et al. (2012) showed that in combination with other methods like the imaging-based ones a complete description of microbial behavior in solid (food) systems can be obtained.

## CONCLUSION

Bacteria in solid (food) systems are known to grow forming colonies. This structured environment constraints cell mobility as well as nutrient and oxygen availability, giving rise to pH and oxygen gradients, due to diffusion limitations inside and around the colonies. Since studies of bacterial behavior have been mainly performed in liquid systems with planktonic cells, it comes of outmost importance to study colony growth dynamics in solid (food) systems, for an effective design of food safety assurance systems. Traditional techniques like viable plate counting, have widely contribute to increase the knowledge about bacterial growth in solid systems. However, these techniques provide information of the overall population but not of individual colonies and additionally, their invasive character requires the consequent sample discard. In order to overcome these disadvantages, new non-invasive techniques are being developed. Most of these novel techniques are based on processing microscopic images obtained from monitoring bacterial colony growth. From image processing, information regarding, e.g., morphology, the colony radius and colony area is gathered and related to bacterial growth. Other techniques based on OD measurements and microcalorimetry, provide information of colony appearance and evolution. Additionally, microcalorimetry has been shown to provide similar information to viable plate counting, thus, power–time curves can be used together with other measurements. Nevertheless, these techniques also show some disadvantages, i.e., the extended time required for data analysis with OD-based techniques and the limited number of experimental conditions that can be assessed with the gel-cassette, etc. In order to overcome these disadvantages, novel techniques are usually combined with viable plate counting. Further research is required to get more accurate knowledge about colony growth dynamics.

### Conflict of Interest Statement

The authors declare that the research was conducted in the absence of any commercial or financial relationships that could be construed as a potential conflict of interest.
